# Dynamic effect of electromagnetic induction on epileptic waveform

**DOI:** 10.1186/s12868-022-00768-y

**Published:** 2022-12-19

**Authors:** Yuqin Sun, Yuting Chen, Hudong Zhang, Yuan Chai

**Affiliations:** School of Mathematics and Physics, Shanghai University of Electric Power, Shanghai, 201306 China

**Keywords:** Electromagnetic induction, Spike and wave discharges, Coupled model, Hopf bifurcation, Absence seizures

## Abstract

**Background:**

Electromagnetic induction has recently been considered as an important factor affecting the activity of neurons. However, as an important form of intervention in epilepsy treatment, few people have linked the two, especially the related dynamic mechanisms have not been explained clearly.

**Methods:**

Considering that electromagnetic induction has some brain area dependence, we proposed a modified two-compartment cortical thalamus model and set eight different key bifurcation parameters to study the transition mechanisms of epilepsy. We compared and analyzed the application and getting rid of memristors of single-compartment and coupled models. In particular, we plotted bifurcation diagrams to analyze the dynamic mechanisms behind abundant discharge activities, which mainly involved Hopf bifurcations (HB), fold of cycle bifurcations (LPC) and torus bifurcations (TR).

**Results:**

The results show that the coupled model can trigger more discharge states due to the driving effect between compartments. Moreover, the most remarkable finding of this study is that the memristor shows two sides. On the one hand, it may reduce tonic discharges. On the other hand, it may cause new pathological states.

**Conclusions:**

The work explains the control effect of memristors on different brain regions and lays a theoretical foundation for future targeted therapy. Finally, it is hoped that our findings will provide new insights into the role of electromagnetic induction in absence seizures.

## Introduction

Absence epilepsy, characterized by transient disturbance of consciousness, is a kind of generalized non-convulsive epilepsy [1]. As one of the most typical refractory diseases, it has extremely complex manifestations [2]. Clinically, electroencephalogram (EEG) is mainly used to record the discharges of neurons and detect the characteristics of absence seizures [3, 4]. In recent years, with the in-depth study of clinical trials, the evolution of typical to atypical pathological states has been widely concerned [5, 6]. The characteristics of absence epilepsy are no longer limited to spike-and-slow wave discharges of 2–4 Hz, but are subdivided into alternating multiple spike-wave oscillations, tonic oscillations, etc. [7, 8]. However, many dynamic phenomena in recorded EEG are still mysterious. It is still unclear what dynamic mechanisms are hidden behind the diversification of pathological features, and whether the choice of models and different parameters can induce new pathological changes. Therefore, the influence of various random factors on the mechanisms of epilepsy is still worthy of further discussion.

Building biophysical models is the primary task for understanding epilepsy. Many scholars have subsequently discussed the etiology of epilepsy by building different neural field models [9, 10]. In 2014, Taylor built a fully functional thalamocortical model to simulate the system, reappeared the spike and wave discharges (SWDs) phenomenon with practical significance and studied the influence of noise on the dynamic characteristics of the system [11]. In 2017, Fan et al. found that the multi-spike wave discharges phenomenon may be related to fold of cycles bifurcations [12]. In 2019, Wang et al. extended the single model to the two-compartment one-way coupled model and found that the interaction between compartments could increase the occupied area of absence epilepsy by a small margin [13]. In 2020, Zhang et al. introduced a second inhibitory neuron to study the dynamic bifurcation mechanism, and proved that Hopf bifurcation participated in the transition of the system from the steady state to the unstable limit cycle [14]. Although experimental studies in recent years have provided some valuable insights into the pathogenesis of epilepsy, most theorists are limited to the discussion of single model or single parameter. If the model structure or related parameters change, some conclusions may no longer be valid. Therefore, we need to add more factors learn more about epilepsy. It should be emphasized that dynamic analysis is still an effective means to study the expansion and reduction of SWDs.

It is worth noting that electromagnetic induction is an important factor affecting the electrophysiological activities of neurons [15]. Some studies have confirmed that the electromagnetic field mainly interferes with the membrane potential between individual and group neurons through current [16, 17]. Later, some scholars began to pay attention to the relationship between the memristor and epilepsy. Vinaya et al. demonstrated that memristors played a leading role in controlling the absence seizures and contribute to the alleviation of absence seizures [18]. By applying the memristor to PY neurons, Zhao et al. found that absence seizures were not only inhibited but might have the opposite result under electrical radiation [19]. In fact, these two arguments are not contradictory. The difference of conclusions just emphasizes the importance of considering different parameters and models of absence seizures. However, most scholarly studies have only explored the effects of electromagnetic induction on absence seizures, rarely combined with the dynamic mechanism, especially the effects between different neuronal populations have not been adequately investigated. Moreover, the uncertainty of parameters space range and the discussion of the diversity of models are often neglected.

In order to break through these limitations, we design a cortical thalamus network model improved by electromagnetic induction. Our main purpose is to compare the dynamic evolution mechanism between single-compartment model and coupled model. In addition, in order to break the limitations of the model discussion, we further consider selecting the coupling strength between multiple neuron populations as dynamic parameters and expanding the numerical simulation range with adjustable parameters. In “Dynamic changes induced by coupling strength in different models” section, we mainly prove that the coupled model can trigger more discharge states such as 2-spike and wave discharges (2-SWDs) and rapid spike discharges with irregular periodic amplitude. See“Single model under electromagnetic induction” and “Coupled model under electric attraction” sections focus on control effects of different model structures and different connection parameters on absence seizures when applying the memristor. Finally, in the conclusion part, we summarize the simulation results of the experiment. The main contribution of this work is to emphasize that electromagnetic induction is of great significance in the treatment of epilepsy in different brain regions. We find that the different choice of connection parameters may lead to the transition from epileptic state to normal background state, or it may also lead to more complicated pathological state and aggravate the area of absence seizures. In particular, this paper combines bifurcation analysis to explain the rich dynamic phenomena and reveals the differences of the mechanisms involved in different discharge states. This paper provides a new vision for a deeper understanding of electromagnetic induction, hoping to provide new ideas for clinical treatment of epilepsy.

## Models and methods

### Network definition and topological analysis

**Fig. 1:**
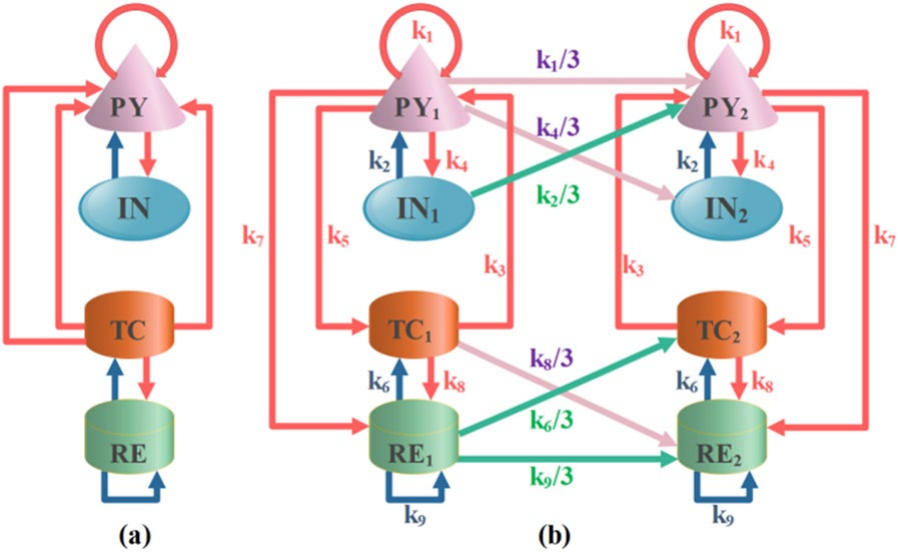
Original and coupled thalamocortical model diagrams. **a** A corticothalamic model consisting of four neuronal populations. **b** Two-compartment coupling topology PY_*i*_, IN_*i*_, TC_*i*_, RE_*i*_ (*i* = 1, 2). The neural population interactions of PY_*i*_, IN_*i*_, TC_*i*_, RE_*i*_ (*i* = 1, 2) constitute the coupling model. Red and dark blue arrows indicate excitatory and inhibitory connections. Purple and green arrowhead lines represent excitatory and inhibitory connectivity of the left compartment to the right compartment

Biophysical computational models are an important way to rapidly recognize and understand absence epilepsy [20, 21]. Many scholars have proposed hypotheses for the abnormal discharges of epilepsy, and good research results have been achieved based on the cortical-thalamic network model [22, 23]. The original Taylor model as shown in Fig. 1a, the pink cone-shaped structure represent the excitatory pyramidal neurons PY, and the blue elliptical structure represent the inhibitory neurons IN, which together form the cortical part [24]. The orange and green cylinders represent TC (specific relay nucleus) and RE (thalamic reticular nucleus), which together form the thalamic portion [25].

All neurons are not separate individuals, and neuronal populations interconnect and interact with each other [26, 27]. To investigate the effect of spatial topology on different neuronal populations, we studied the complex structure as shown in Fig. 1b. In this paper, we introduce the electromagnetic induction mechanism into the coupled cortical thalamus model proposed by Wang et al. [13]. The improved model can be described by the following differential equations [12, 13, 19, 28]:

1 $$\frac{{dPY_{1} }}{dt} = \,(\varepsilon_{py} - PY_{1} + k_{1} F[PY_{1} ] - k_{2} F[IN_{1} ] + k_{3} F[TC_{1} ] - k_{0} \rho (\phi_{1} )PY_{1} ) \, \tau_{1} ,$$

2 $$\frac{{dIN_{1} }}{dt} = (\varepsilon_{in} - IN_{1} + k_{4} F[PY_{1} ]) \, \tau_{2} {, }$$

3 $$\frac{{dTC_{1} }}{dt} = (\varepsilon_{tc} - TC_{1} + k_{5} F[PY_{1} ] - k_{6} G[RE_{1} ]) \, \tau_{3} {, }$$

4 $$\frac{{dRE_{1} }}{dt} = (\varepsilon_{re} - RE_{1} + k_{7} F[PY_{1} ] + k_{8} G[TC_{1} ] - k_{9} G[RE_{1} ]) \, \tau_{4} {, }$$

5 $$\begin{aligned} \frac{{dPY_{2} }}{dt} = \,(\varepsilon_{py} - PY_{2} + k_{1} F[PY_{2} ] - k_{2} F[IN_{2} ] + k_{3} F[TC_{2} ] \\ - k_{0} \rho (\phi_{2} )PY_{2} ) \, \tau_{1} + \frac{{k_{1} }}{3}F[PY_{1} ] - \frac{{k_{2} }}{3}F[IN_{1} ], \\ \end{aligned}$$

6 $$\frac{{dIN_{2} }}{dt} = (\varepsilon_{in} - IN_{2} + k_{4} F[PY_{2} ]) \, \tau_{2} \, + \frac{{k_{4} }}{3}F[PY_{1} ],$$

7 $$\frac{{dTC_{2} }}{dt} = (\varepsilon_{tc} - TC_{2} + k_{5} F[PY_{2} ] - k_{6} G[RE_{2} ]) \, \tau_{3} - \frac{{k_{6} }}{3}G[RE_{1} ],$$

8 $$\begin{gathered} \frac{{dRE_{2} }}{dt} =\, (\varepsilon_{re} - RE_{2} + k_{7} F[PY_{2} ] + k_{8} G[TC_{2} ] - k_{9} G[RE_{2} ]) \, \tau_{4} + \frac{{k_{8} }}{3}G[TC_{1} ] - \frac{{k_{9} }}{3}G[RE_{1} ] \hfill \\ \, \hfill \\ \end{gathered}$$

9 $$\, \frac{{d\phi_{i} }}{dt} = \lambda_{1} PY_{i} - \lambda_{2} \phi_{i} ,$$

10 $$\rho (\phi_{i} ) = \alpha_{1} + 3\beta_{1} \phi_{i}^{2} .$$

*ε*_*py*_, *ε*_*in*_, *ε*_*tc*_, *ε*_*re*_ are additive constants, *τ*_1_–*τ*_4_ denote different time scales. *k*_1_-*k*_9_ represent the strengths of connections between different neuronal populations. *F*[.] and *G*[.] are activation functions, which are mainly used to describe the cortical subsystem and thalamic subsystem. *F*[*x*] = 1/(1 + *ε*^−*x*^) where *x* = PY_*i*_, IN_*i*_, TC_*i*_, RE_*i*_ (*i* = 1, 2), and *ε* represents the steepness of sigmoid function *F*[*x*]. *G*[*x*] = *ax* + *b* where *x* = TC_*i*_, RE_*i*_ (*i* = 1, 2) [15, 29].

### Induction of electrical stimulation

We use memristor to realize the coupling relationship between average magnetic flux and average membrane potential, and propose a more reliable thalamocortical model with electromagnetic induction [15, 30, 31]. Due to the dominant role of pyramidal neurons, we only consider the electromagnetic induction of pyramidal neurons. In Eq. (1), *λ*_1_ and *λ*_2_ are related to electromagnetic induction, and the terms *λ*_1_PY_*i*_ and *λ*_2_PY_*i*_ (*i* = 1, 2) denote the effect of electromagnetic induction and self-induction, respectively. *ϕ*_*i*_ (*i* = 1, 2) means that the average magnetic flux is the magnetic flux passing through the cell membrane. *ρ*(*ϕ*_*i*_) is the coupling strength between the membrane potential of neurons and magnetic flux. It is a memristor controlled by magnetic flux, which is equivalent to memory conductance. *ρ*(*ϕ*_*i*_) is often described by Eq. (10), where *α*_1_, *β*_1_ are fixed parameters. According to Faraday's law of electromagnetic induction and the description of memristor, the fluctuation of membrane potential will produce induced current, which is expressed by the term *k*_0_*ρ*(*ϕ*_*i*_)PY_*i*_ in Eq. (1). *k*_0_ represents the feedback gain of the average magnetic flux. *k*_0_ = 0 or 0.5 is used to compare the difference between no average magnetic flux and electromagnetic induction interference.

### Simulating method

This paper uses MATLAB environment to simulate. The fourth-order Runge–Kutta method is used to solve the delay differential equations of four neurons in the left compartment and right compartment in the model. The simulation time is set to 30 s long enough, and the fixed time step of numerical integration is 0.05 ms. Most of the parameters in this paper are taken from previous experimental studies, and the values of all parameters are shown in Table 1 [13, 19, 32]. In order to see more abundant dynamic phenomena, the connection strengths are set within a certain reasonable range (*k*_1_–*k*_9_). The value of *k*_0_ is generally 0 or 0.5, which indicates whether the system is disturbed by electromagnetic field. In addition, we describe the macroscopic dynamics of cortex by using extreme value diagrams and dominant frequency diagrams. The main frequency is simulated by fast Fourier transform. Finally, we obtain the bifurcation results of system dynamics using the continuation package AUTO in XPPAUT software.

## Numerical results

### Dynamic changes induced by coupling strength in different models

It has been confirmed that sensory movement at the individual level is not caused by the emission of electricity from individual neurons, but by the collective behavior of many neurons in the cortex, thalamus and spinal cord in the brain system [33, 34]. Therefore, we no longer limit our discussion to a single pathway within or outside the thalamus, but focus on multiple excitatory inputs and inhibitory projections from the cortex and thalamus [35, 36]. In this section, we selected eight coupling strengths *k*_*i*_ (*i* = 1, 2, 3, 5, 6, 7, 8, 9) between cortex and thalamus as key parameters to explore the dynamic changes of epileptic waveforms in single and coupled models. Our main purpose is to find the dynamic relationship of the system by studying the changes between different single pathways and to pave the way for comparing the dynamical changes induced by electromagnetic induction.

**Fig. 2:**
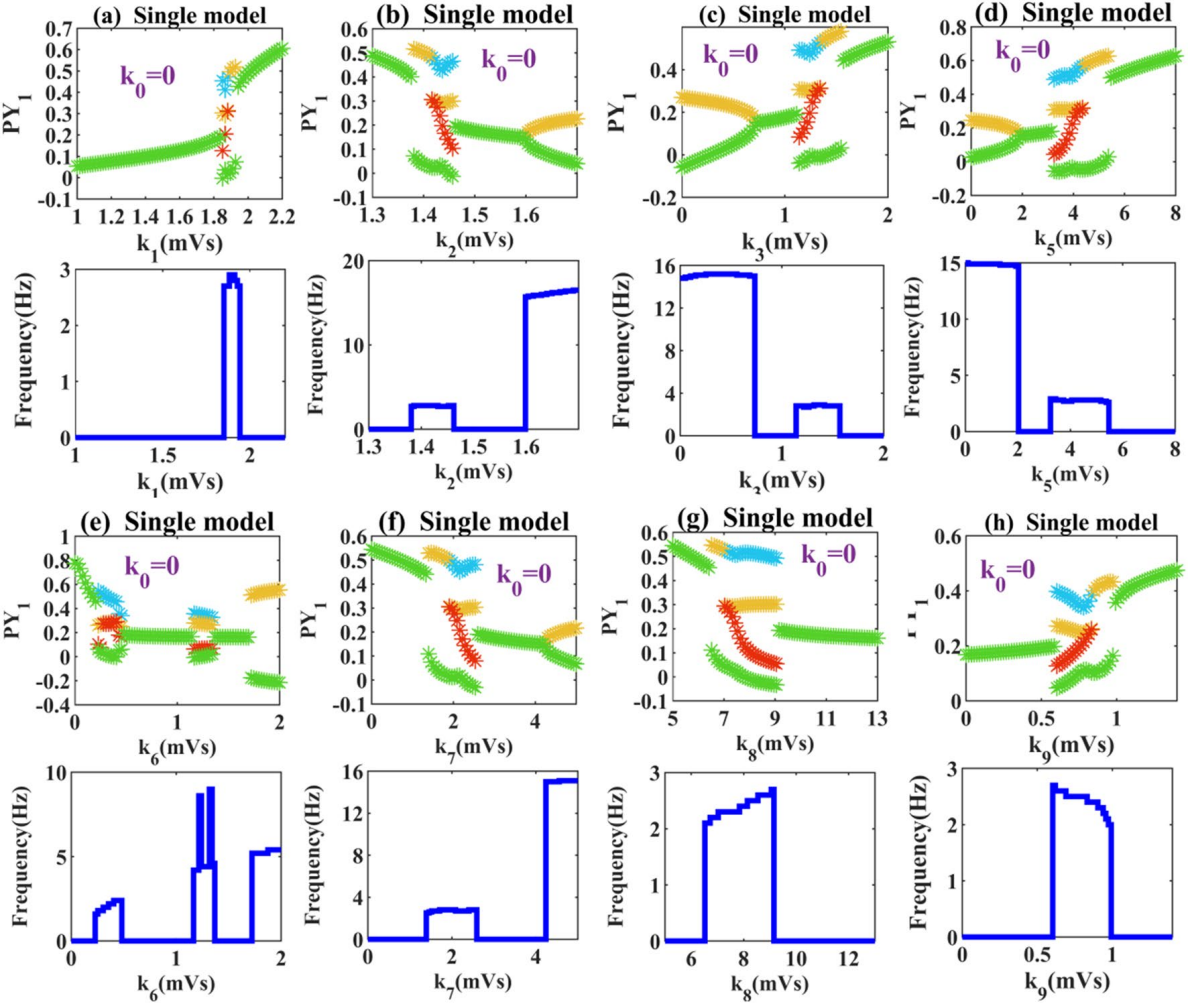
Extreme values of PY_1_ and the corresponding dominant frequency diagrams: when these parameters **a** *k*_1,_ **b** *k*_2_, **c** *k*_3_, **d** *k*_5_, **e** *k*_6_, **f** *k*_7_, **g** *k*_8_, and **h** *k*_9_ are varied within the set range, the state of the system will change to some extent. **a**–**d** The system can be observed in high and low saturated states and SWDs state. **e**–**h** The system adds tonic oscillations with a frequency around 15 Hz

**Fig. 3:**
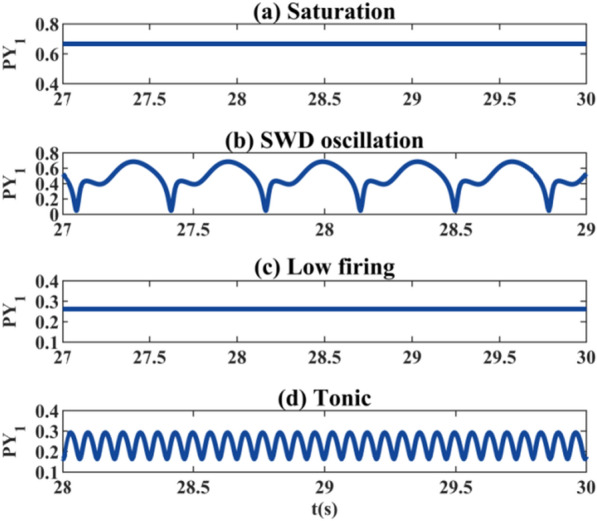
Time series diagram of PY_1_ under single chamber model. Corresponding to Fig. 2f, select the parameters **a** *k*_7_ = 1, **b** *k*_7_ = 2, **c** *k*_7_ = 3, and **d** *k*_7_ = 4.5

First, we get rid of the electric radiation and simply draw the bifurcation diagrams in the single-compartment model. We can see that in Fig. 2a, with the change of bifurcation parameter *k*_1_, the system state continuously transits from low discharge to pathological SWDs and high saturation discharge. Interestingly, the same dynamic transfer mechanisms can be seen in Fig. 2h by adjusting *k*_9_ reasonably. We have verified that by adjusting the connection strengths of the two pathways of cortex self-excitation (*k*_1_) and thalamus self-inhibition (*k*_9_), the absence seizures can be reproduced and disappeared. In Fig. 2b, with the change of the bifurcation parameter *k*_2_, the system exhibits a richer dynamical characteristics. When *k*_2_ ≥ 1.604, the system transitions from a low firing state to a tonic state, which corresponds to a dominant frequency around 16 Hz. In Fig. 2c and d, the initial state of the system is high-frequency tonic discharge. With the increase of synaptic connection strengths (*k*_3_ or *k*_5_), the system changes in three different states. In Fig. 2e we observe more obvious state fluctuations. Not only the lower (0.22 < *k*_6_ < 0.46) coupling strength can initiate SWDs, but also SWDs can be triggered when *k*_6_ > 1.7. Also, a small pathological discharge (1.16 < *k*_6_ < 1.34) appears in the low discharge (0.22 < *k*_6_ < 1.7) region. In Fig. 2f, when *k*_7_ ≥ 1.4, the normal background state of the system is broken and the absence seizure phenomenon occurs. In Fig. 2g, when the coupling strength *k*_8_ is low, the activation level of the RE neuron is small enough to inhibit the TC neuron. So the initial state of the system is high saturated firings. With the increasing of *k*_8_, the activation of some TC neurons is inhibited, which makes the system appear SWDs. When *k*_8_ ≥ 9.152, the system shows low discharge. Here, we take Fig. 2f as an example, select several specific values to draw the discharge diagrams as shown in Fig. 3, high saturated state (Fig. 3a), SWDs state (Fig. 3b), low saturated state (Fig. 3c) and tonic state (Fig. 3d).

**Fig. 4:**
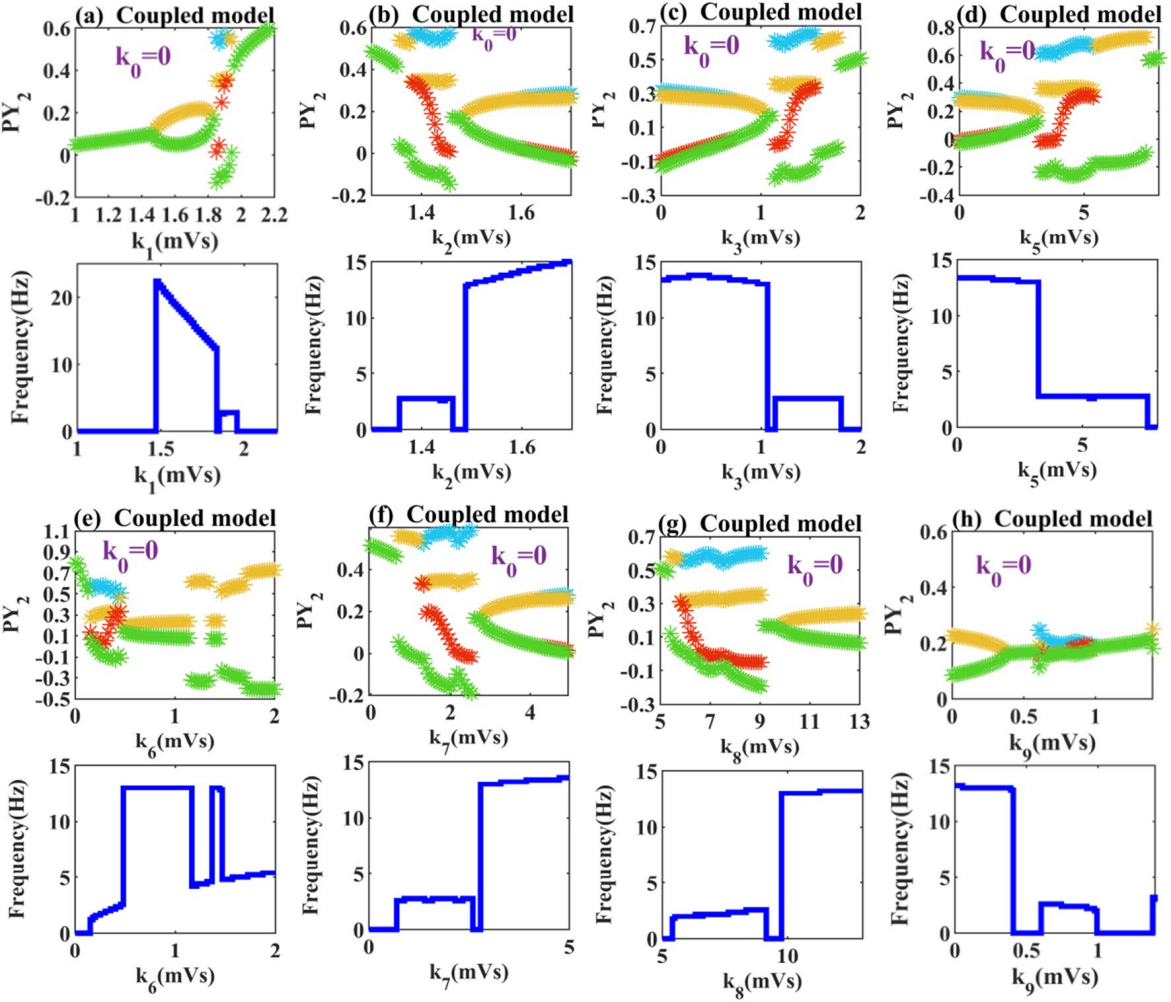
Extreme values of PY_2_ and the corresponding dominant frequency diagrams: when these parameters **a** *k*_1_, **b** *k*_2_, **c** *k*_3_, **d** *k*_5_, **e** *k*_6_, **f** *k*_7_, **g** *k*_8_, and **h** *k*_9_ are varied within the set range, the state of the system will change to some extent. Here, *k*_0_ = 0

Next, in order to observe what effect the interaction between compartments will have on absence epilepsy. We set the same parameter range for the connection strength of different pathways as the single compartment model, drawing the bifurcation diagrams of the coupled model. As shown in Fig. 4a, with the increase of coupling strength, the steady state is broken when *k*_1_ > 1.48, which changes the model from low firing oscillation to tonic oscillation. And as the inhibition of left compartment to right compartment increases, the dominant frequency corresponding to tonic oscillation gradually decreases. In Fig. 4b–f, we find that the coupled model is prone to transform the tonic oscillation into a fast spike discharge with periodic up and down amplitude fluctuations. Although the waveform changes, the corresponding fluctuation frequency is basically unchanged.

In Fig. 4e, when the coupling strength *k*_6_ is large enough (*k*_6_ > 1.7), the system transitions from SWDs to clonic discharge with a frequency of about 5 Hz. In Fig. 4g, a smaller coupling strength (*k*_8_ = 5.4) makes the system transition from high saturation state to large SWDs state. When *k*_8_ > 9.8, the low firing oscillation of the system disappears and the system behaves as a tonic state. In Fig. 4h, with the increased inhibition of RE neurons at around *k*_9_ = 0.6 the system shows 2–4 Hz spike and slow wave discharge. Until the excessive inhibition of the left compartment on the right compartment increases the connection strength of *k*_9_ to about 1, the pathological state disappears and the system shows low discharge with the frequency of 0 Hz.

Macroscopically, the effects of the coupled model for completely different populations of pathways share a common feature: the expansion of pathological area. We find that the right compartment, whether stimulated by excitability or inhibited projection of left compartment, show the result that the normal background was easily transformed into atypical pathological area. It is particularly noted that in Fig. 4c, d, f, and g the pathological state covers almost more than 80% of the area within the parameters we set. It can be seen that the discussion of complex model is an important part of epilepsy research.

**Fig.5:**
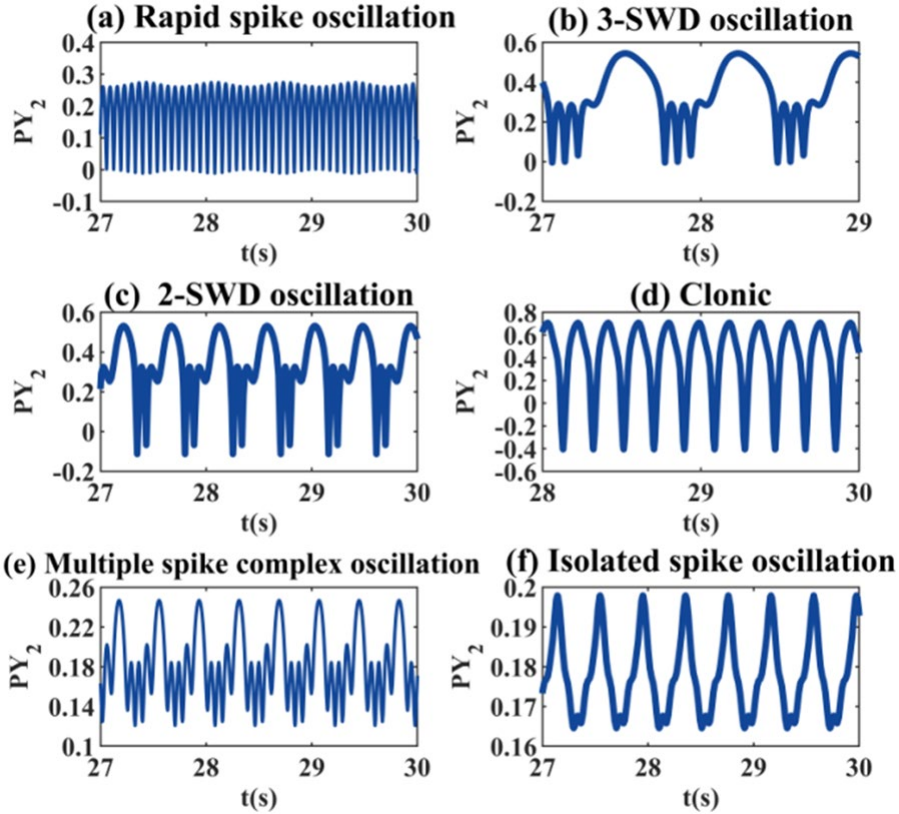
Time series plot of PY_2_ under the coupled model. The other parameters are shown in the graphs, and the selected parameters **a** *k*_2_ = 1.65, **b** *k*_6_ = 0.21, **c** *k*_6_ = 0.37, **d** *k*_6_ = 1.8, **e** *k*_9_ = 0.62, and **f** *k*_9_ = 0.8

Next, we select several specific parameters in different states to draw the discharge diagram (Fig. 5), and find the special pathological states that do not appear in a single model. As shown in Fig. 5a, the system experience a rapid sharp wave oscillation with a frequency of about 15 Hz and the amplitude exhibits periodic fluctuations when *k*_2_ = 1.65. In Fig. 5b, the system breaks through the typical SWDs and the 3-SWDs around 2 Hz appears as *k*_6_ = 0.21. As shown in Fig. 5c, the system shows 2-SWDs at 2–3 Hz when *k*_6_ = 0.37. The appearance of multiple spike wave discharges often predicts the onset of spasm, which is the main waveform of myoclonic epilepsy [37]. In Fig. 5d, when *k*_6_ = 1.8 the system shows a low-frequency clonic oscillation of about 5 Hz, which has a high amplitude between 0.8 and 0.9. In Fig. 5e and f, we plot the special discharge associated with *k*_9_. When *k*_9_ = 0.62, the system shows multiple spike complex oscillation with increasing periodic amplitude and frequency essentially greater than 13 Hz. When *k*_9_ = 0.8, the system shows isolated spike oscillation. These two waveforms are mostly related to paroxysmal epilepsy.

### Single model under electromagnetic induction

It has been shown that fluctuations in the membrane potential of neurons can have a significant impact on the distribution of electromagnetic fields, causing changes in magnetic flux and electromagnetic induction across the cell membrane [38, 39]. Therefore, it is crucial to consider the effect of electromagnetic induction on neuronal activity in the model. Memristor is considered as a perfect device to simulate neural synapses, and the artificial neural network using memristor to simulate neural synapses is called memristor neural network [40, 41]. In this section, the classical single cortical thalamus model is improved by using the memristor model with magnetic flux variable, and the main purpose is to use the memristor to regulate the electrophysiological activities of neurons. We investigate the influence of electromagnetic radiation within the nervous system on the dynamic characteristics of different neuron populations, and compare it with a single model free from electrical radiation.

**Fig. 6:**
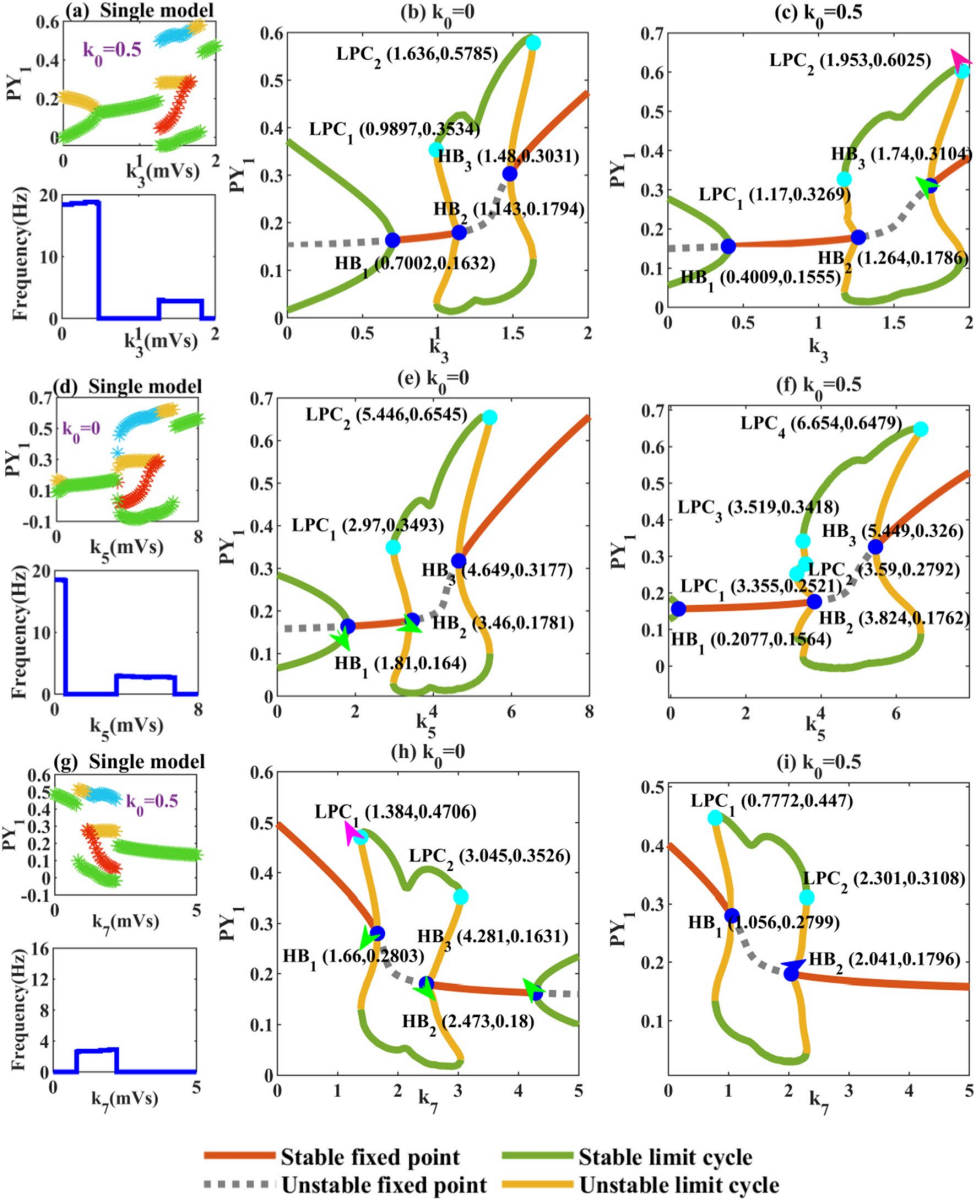
Absence seizures caused by changes in the intensity of excitatory stimulation between the cortex and the thalamus. Left panel: draw extreme value diagram and corresponding main frequency diagram, middle panel: dynamic analysis diagram without memristor interference, and right panel: dynamic analysis diagrams of interaction with memristor. **a**–**c** adjust the connection strength of TC to PY between 0 and 2, **d**–**f** scan *k*_5_ from 0 to 8, and **g**–**i** scan *k*_7_ from 0 to 5. The dark blue and light blue solid dots represent hopf bifurcations (HB_*i*_, *i* = 1, 2, 3) and fold of cycles bifurcations (LPC_*j*_, *j* = 1, 2, 3, 4)

We first explore the dynamic effects of changes in the connection parameters (*k*_3_, *k*_5_, *k*_7_) of cortico-thalamic interactions in a macroscopic sense on the system separately. It can be clearly seen from the bifurcation diagram of Fig. 6a that under the combined effect of *k*_3_ and memristor, the system can transition from tonic oscillation to low firing oscillation with less excitatory stimulus of TC to RE. Compared with Fig. 2c, the high saturated firings when 1.57 < *k*_3_ < 1.67 is converted into SWDs, and the absence seizures of the system are aggravated. In Fig. 6b and c, we observe that electric radiation affects the early generation of HB_1_ point, which means that the tonic state induced by limit cycle ends prematurely. When 0.4009 ≤ *k*_3_ ≤ 1.264, the system transits to a wider range of monostable state. Meanwhile, the electric radiation delays the generation of HB_2_ and HB_3_ points, which is also the reason for the delay and aggravation of SWDs phenomenon in the system.

Compared with Fig. 6e, we can clearly see in Fig. 6f that electromagnetic induction induces more bifurcation mechanisms. In detail, the HB_1_ point is shifted from 1.81 to the left to 0.2077, and the tonic state was well suppressed by the reduction of limit cycle range. When 0.2077 < *k*_5_ < 3.355, the limit cycle disappears and the system converges to a stable fixed point, which corresponds to the low firing oscillation in Fig. 6d. When 3.355 < *k*_5_ < 3.519, the fold of cycles bifurcation (LPC_1_) appears, making the system transition from monostable to bistable state. When 3.519 < *k*_5_ < 3.59, two stable limit loops appear, making the system transition to tristable state. We suggest that the appearance of 2-SWDs in Fig. 6d is closely related to the generation of LPC_1_, LPC_2_. LPC_1_ gradually disappears after the start of LPC_3_ and the system becomes bistable state (3.59 < *k*_5_ < 3.824). When *k*_5_ = 3.824, the appearance of HB_2_ changes the state of the fixed point from stable to unstable, and the system enters a monostable state. When 5.449 < *k*_5_ < 6.654, HB_3_ makes the fixed point return to a stable state, and the system enters the bistable region again. When 6.654 < *k*_5_ < 7, the fold of cycles bifurcation disappears and the system returns to the monostable state.

In Fig. 6g, the system is reduced from the original five states to four states under the action of the memristor, and the tonic state disappears. Comparing Fig. 6h and i, it is not difficult to find that the fold of cycles bifurcations (LPC_1_ and LPC_2_) of the system advance and the Hopf bifurcation point decreases. Therefore, the system transits from high saturated firings to SWDs with smaller connection strength and the supercritical bifurcation induced by HB_3_ disappears. That is, in Fig. 6h, when *k*_7_ > 2.041, the solutions of the system converge to stable fixed points. To sum up, electric radiation promotes the early generation of pathological state. Fortunately, in Fig. 6a, d, and g, we all see that the tonic oscillation of the system under electric attraction is reduced. In Fig. 6g, the tonic state disappears.

**Fig. 7:**
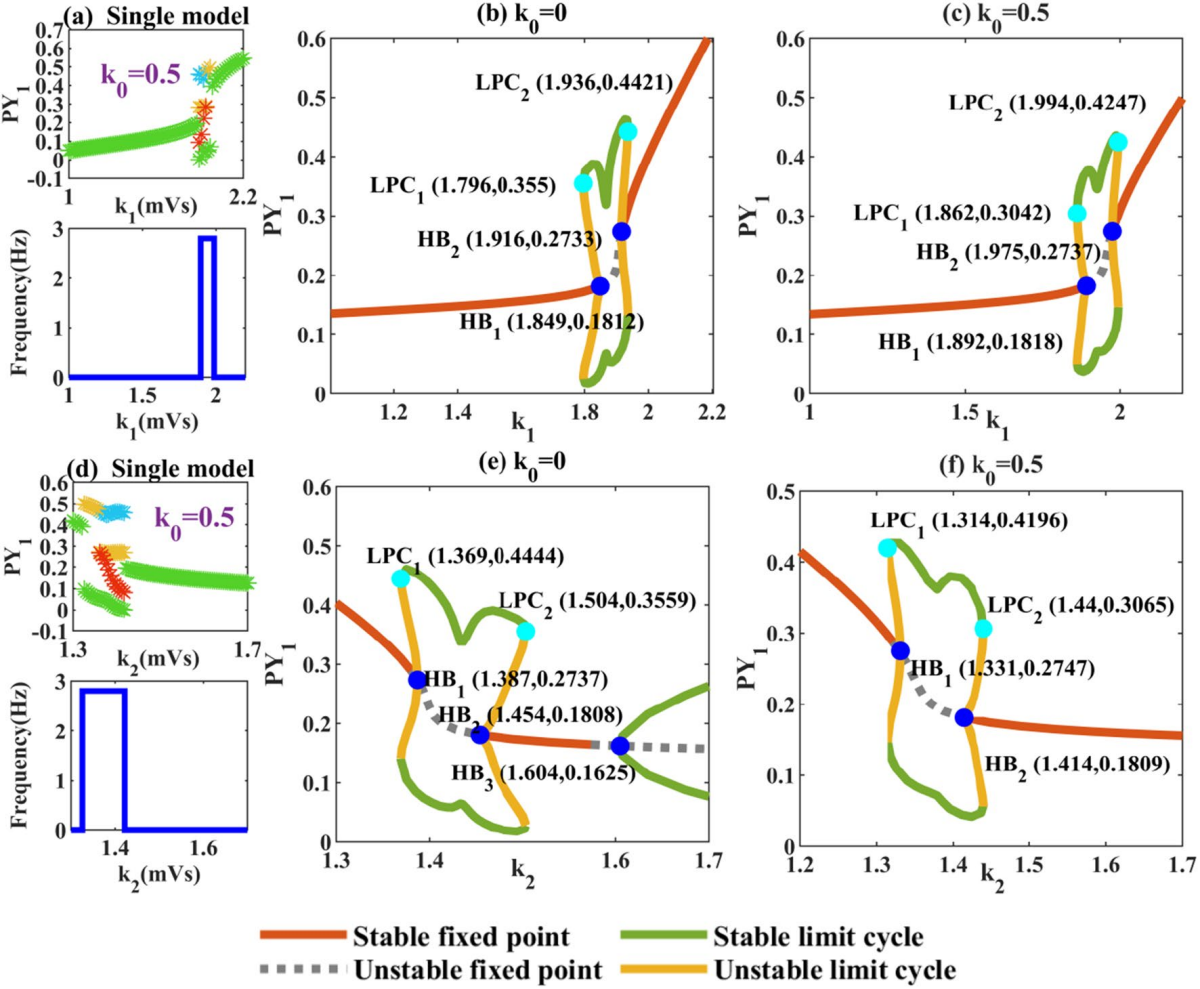
Dynamic changes caused by the change of coupling strength of PY-PY and IN-PY. **a**, **d** Extreme value diagram and corresponding dominant frequency diagram of PY_1_ related to parameters *k*_1_, *k*_2_. **b**, **e** Dynamic analysis diagram of parameters *k*_1_, *k*_2_ without electromagnetic interference. **c**, **f** Dynamic analysis diagram of *k*_1_, *k*_2_ under electromagnetic radiation

Then we focus on the internal function of cortex and thalamus. In order to independently discuss the influence of electrical stimulation target selection on the neural network, *k*_0_ is also set to 0.5. Then we focus on *k*_1_ and *k*_2_, and draw bifurcation diagrams as shown in Fig. 7a and d. For the cortical self-excitation pathway PY-PY, the function of memristor is not obvious. The role of the memristor is not obvious. The dynamic transfer mechanism of the system does not change and shows a larger range of the region of low saturated firing under electrical attraction compared to Fig. 4a. In Fig. 7b and c, we find that when the degree of cortical self-excitation is not high, the system will always converge to a stable fixed point, that is, it shows low saturaed firings. With the increasing degree of cortical self-excitation the system first transits to the bistable region between LPC_1_ and HB_1_, because the appearance of HB_1_ transits to the monostable region. Then a second bistable region appears between HB_2_ and LPC_2_. Finally, the system returns to monostable state with the disappearance of limit cycle.

In addition, we can observe that low and high saturated state can coexist with SWDs (between HB_1_ and LPC_1_, and between HB_2_ and LPC_2_). In Fig. 7d, the enhancement of the inhibition from IN to PY can make the system transition from the initial high saturated state to the pathological SWDs state, and can also make the system transition from absence seizures to low saturated state. As shown in Fig. 7e and f, we can also observe the coexistence of steady state and SWDs state. But the unstable limit cycle generated at HB_3_ can not coexist with electromagnetic induction of a certain intensity. So the number of Hopf bifurcation points is reduced to two, and finally the system transits to the monostable region under the electric attraction, that is, it shows a low saturated firings of 0 Hz.

**Fig. 8:**
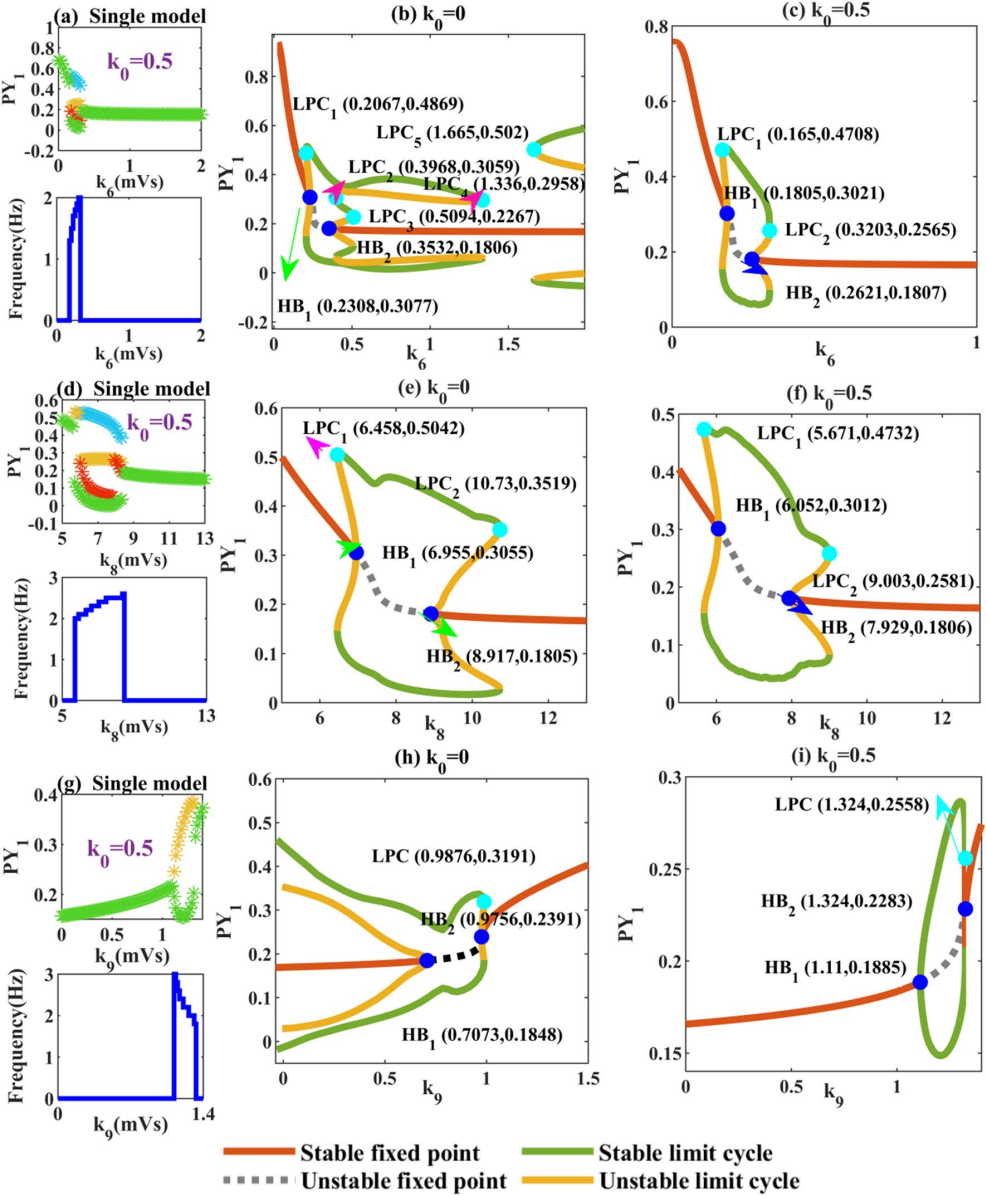
Absence seizures caused by the coupling strengths of TC and RE pathways. **a**, **d**, **g** Time series diagrams of PY_2_ in hypothalamus of electric radiation. **c**, **f**, **i** Dynamic analysis diagrams under the action of memristor and getting rid of memristor **b**, **d**, **h**. HB_*i*_ (*i* = 1, 2) represents Hopf bifurcations, LPC_*j*_ (*j* = 1, 2) represents fold of cycles bifurcations

For the neural population in thalamus, we mainly focus on *k*_*i*_ (*i* = 6, 8, 9). We can see that, as shown in Fig. 8a, the memristor eliminates 2-SWDs, SWDs (> 4 Hz) and tonic that appear in Fig. 2e.With the increasing inhibition of RE on TC, the system only changes in three dynamic states. Furthermore, we find in Fig. 8b that there is a region where low saturated firing and SWDs coexist between Hopf bifurcations and fold of cycles bifurcations (LPC). At the same time, a tristable region is found between LPC_2_ and LPC_3_. In Fig. 8c, the amplitude of the limit cycle increases with the inhibition of RE neurons, and the fold of cycles bifurcation converges to fixed points (*k*_6_ > 0.23). In Fig. 8d, the joint modulation of electromagnetic induction and *k*_8_, the system changes from the original four states to five states (5 ≤ *k*_8_ ≤ 13). That is, the emergence of 2-SWDs. We have learned that the formation of multi-spike discharges is closely related to multi-fold of cycle bifurcations. So we draw the dynamic bifurcation diagrams as shown in Fig. 8d and e. With the enhancement of excitability from TC to RE, the system changes from monostable to bistable region with fold of cycles bifurcation (LPC_1_).The unstable HB_1_ point appear earlier, which make the system return to monostable ahead of time. Then, between LPC_2_ and HB_2_, the system transits to the bistable region composed of limit cycle and stable points, and 2-SWDs appears between them. Until the limit cycle disappears, the system converges to stable fixed points. In Fig. 8g, the memristor has played a very good role. With the increase of *k*_9_, there is no typical absence seizures in the system. When 1.11 ≤ *k*_9_ ≤ 1.31, clonic oscillation with stepwise decreasing frequency of 2–3 Hz appears in the system. In Fig. 8h, slow wave oscillations occurs between LPC and HB_2_ until the limit cycle disappears and clonic oscillations ends. In Fig. 8i, the stability of the fixed point of the system at HB_1_ point disappears. The appearance of fold of cycles bifurcations are also responsible for the slow-wave oscillation generation. To sum up, we find that the system has a good therapeutic effect on atypical pathological state such as tonic state under the joint action of memristor and connection strengths (*k*_2,_ *k*_3_, *k*_5_, *k*_7_). It is worth noting that the parameter *k*_9_ shows a more effective inhibitory effect on absence seizures than other connection parameters.

### Coupled model under electric attraction

The human brain is a very complex neural network. There are tens of billions of nerve cells in the cortex, also known as neurons, each of which can interact with information by connecting other neurons through thousands of synapses [42]. Therefore, the connection of brain network and the change of topology of brain network are the key factors that affect the cerebral cortex system [43, 44]. We have realized from “Dynamic changes induced by coupling strength in different models” Section that the coupled model triggers many states that do not exist in the single-compartment model, which makes the dynamic mechanism of the system more complicated. The role of the memristor in the simple single-compartment model has been understood, and how it affects the diverse dynamical characteristics arising from the more complex two-compartment model needs to be investigated in more depth. Here, we also introduce electromagnetic induction into the coupled model, and discuss in detail the changes of brain activity caused by the arrival of memristors.

**Fig. 9:**
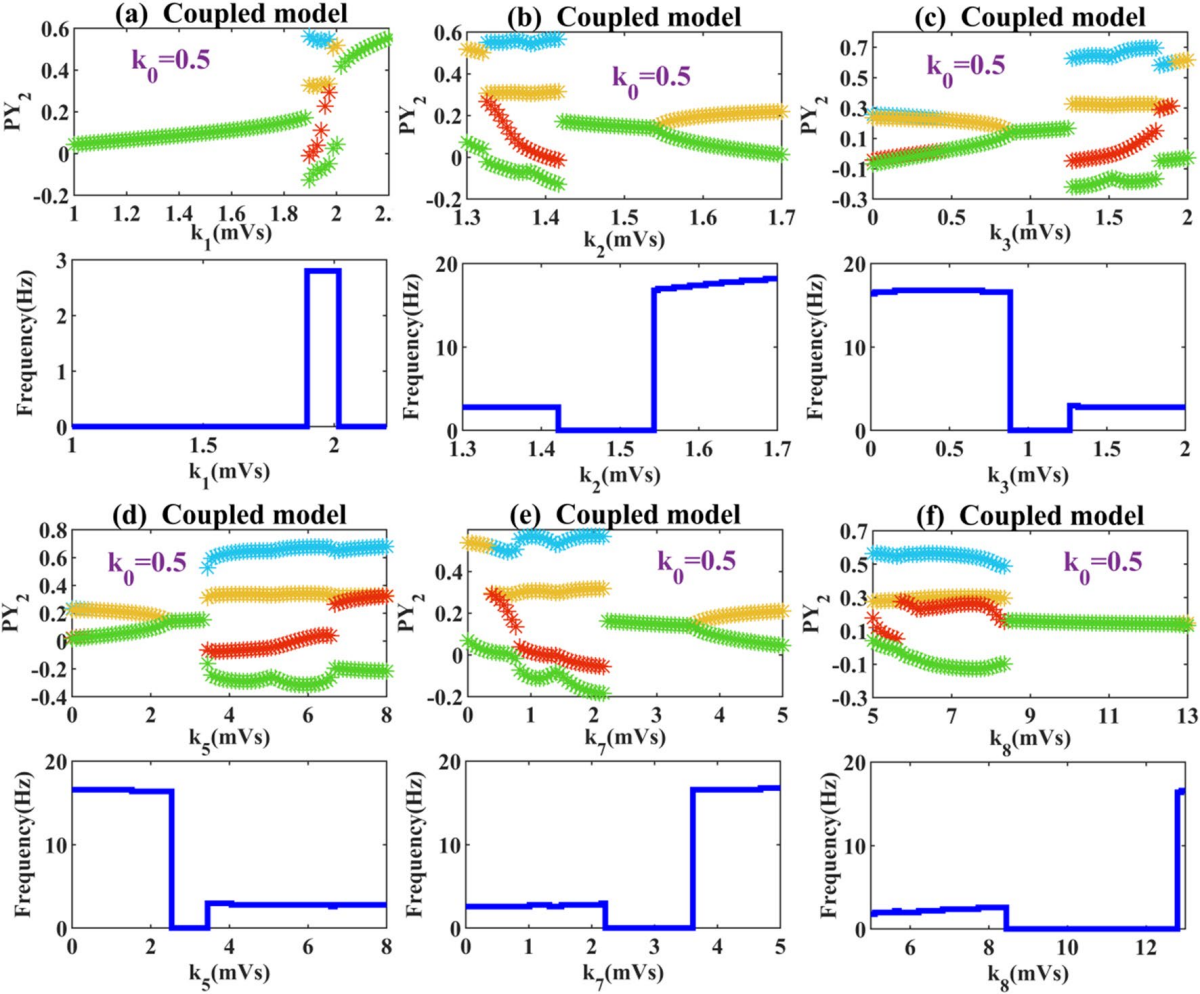
Bifurcation diagrams and corresponding frequency diagrams of different pathways under electric radiation: when these parameters **a** *k*_1,_ **b** *k*_2_, **c** *k*_3_, **d** *k*_5_, **e** *k*_7_, and **f** *k*_8_ are varied within the set range, the state of the system will change to some extent. Here, *k*_0_ = 0.5

In Fig. 9a, we see that the system mainly shows three state transitions with the increase of *k*_1_. Compared with Fig. 4a, we find that the tonic state was completely transformed into low firing oscillation. Similarly, it can be observed in Fig. 9f that the system exhibits only a single low firing oscillation when *k*_8_ > 9. In Fig. 9b, under the combined action of electromagnetic induction and *k*_2_, the absence seizure occurred in advance. The memristor shows the ability to reduce the rapid spike discharge to tonic, which makes the amplitude of fluctuation stable. The initial state of the system changes from high saturated state to clonic. As *k*_7_ also shows a similar state transition mechanism in Fig. 9e, it will not be explained too much. Compared with Fig. 4c, when *k*_3_ > 1.8 in Fig. 9c, the system is unable to convert pathological state into high saturated state. Similarly, in Fig. 9d, when *k*_5_ = 8, the original high saturated firings of the system was replaced by SWDs due to the existence of memristor. This also shows that the memory resistor will interfere with the information exchange between cortex and thalamus to a certain extent.

**Fig. 10:**
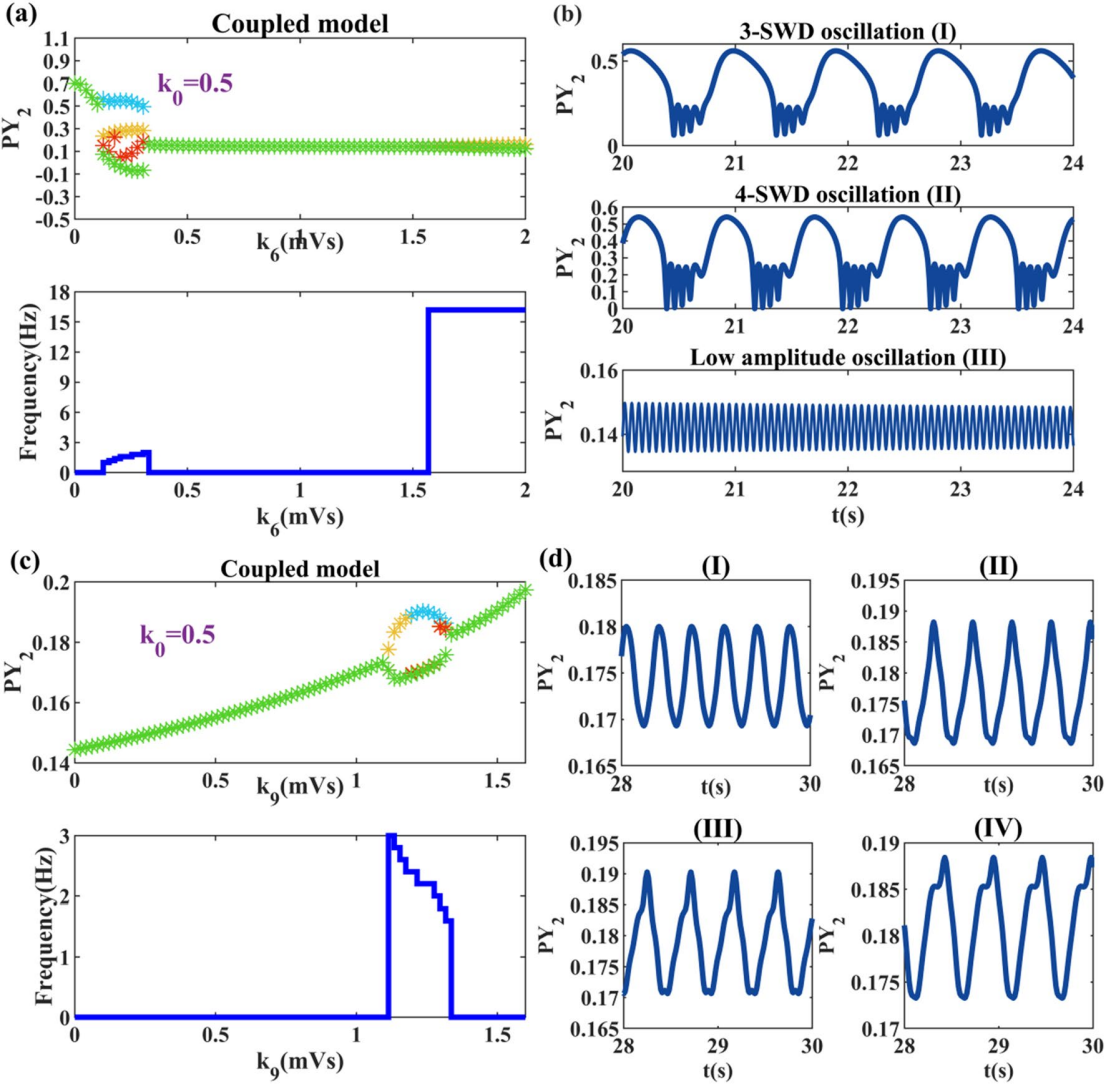
Two inhibitory pathways within thalamus. **a**, **c** Time series diagram and corresponding main frequency diagram induced by pathways *k*_6_ and *k*_9_ under electric radiation. **b** Special discharge diagrams with *k*_6_ = 0.13(I), *k*_6_ = 0.17(II), *k*_6_ = 1.7(III). **d** Close-up of different stages of slow wave oscillation: *k*_9_ = 1.12(I), *k*_9_ = 1.18(II), *k*_9_ = 1.24(III), *k*_9_ = 1.29(IV)

Next, we will introduce in detail what changes will be brought when the electromagnetic induction parameter *k*_0_ is fixed and the parameters *k*_6_, *k*_9_ fluctuate within a certain range. From Fig. 10a, we can see that the state distribution of disorder becomes modular. With the increasing inhibition of RE neurons to TC neurons from bottom to top, the system gradually changes from high saturated state to pathological state, and then to a wide range of low firing state. The distribution of different states in Fig. 10c also shows the feature of regionalization. Unlike Fig. 10a, the system pathological states appear in larger connection strengths and the corresponding frequencies show a stepwise decrease. Then we select specific values to plot the waveforms of the simple oscillation transition changes. states (I) to (IV) in Fig. 10d depict the change process of the slow wave oscillation gradually distorted by the combined effect of the memristor and *k*_9_.

**Fig. 11:**
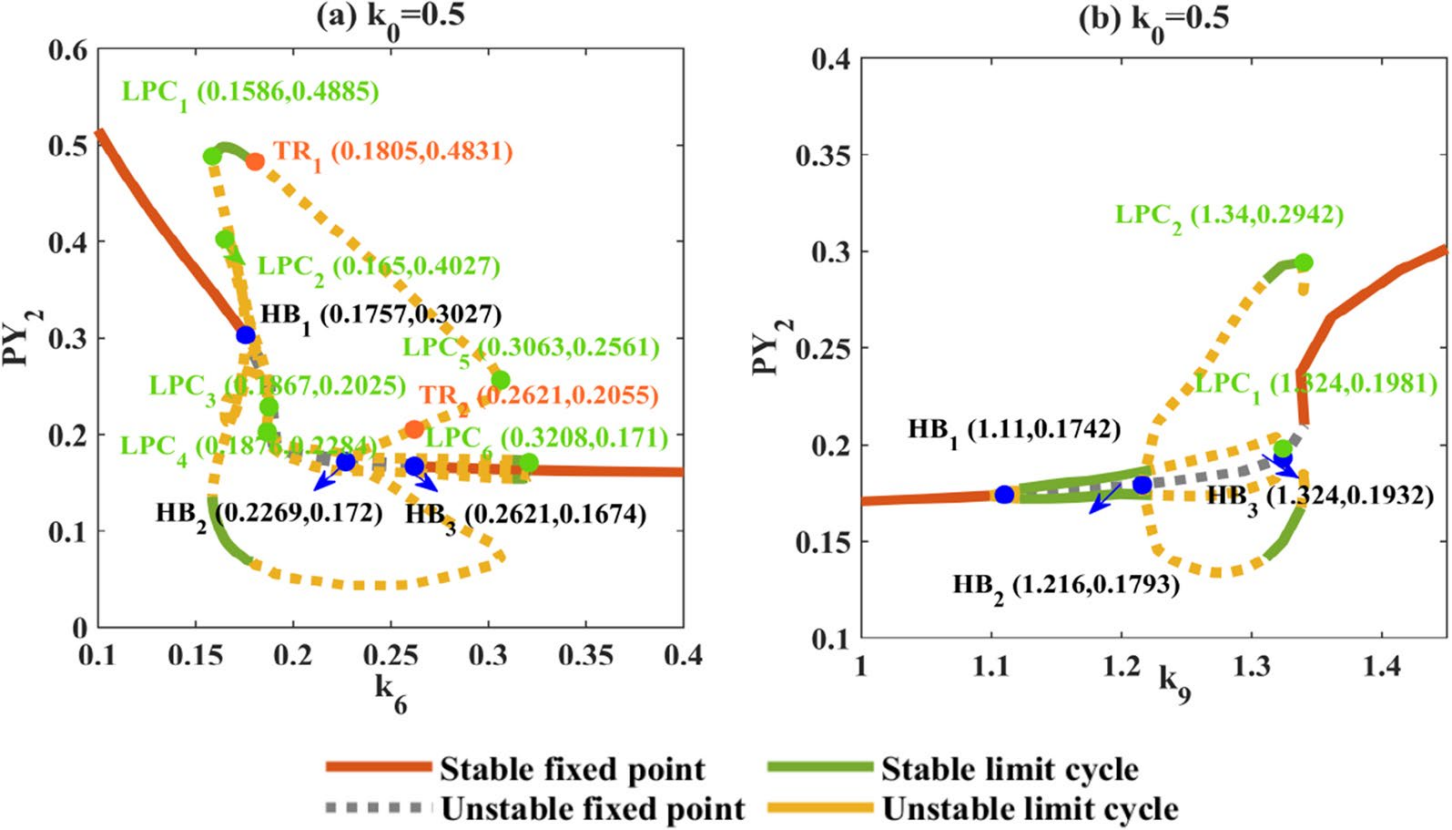
Bifurcation diagram of *k*_6_ and *k*_9_ under electromagnetic interference (**a**, **b**). The solid and dashed lines correspond to the stability and instability of the state. The solid green dots indicate fold of cycles bifurcation (LPC_*j*_, *j* = 1, 2, 3, 4, 5, 6), the solid blue dots indicate Hopf bifurcations (HB_*i*_, *i* = 1, 2, 3), and the solid orange dots indicate Torus bifurcations (TR_*m*_, *m* = 1,2)

Then, we select the range values near the pathological state to draw the dynamic bifurcation diagram. From Fig. 11a, we get that 3-SWDs is accompanied by the fold of cycle bifurcations and 4-SWDs appears near the TR_1_ bifurcation point. Finally, with the disappearance of the limit cycle of LPC_6_, the system enters the monostable state with this line and the low saturated firing appears. As shown in Fig. 11b, with the generation of HB_1_ point the system starts to transition to slow wave oscillation (I) and slowly evolves into waveform (II) with a stable limit cycle. Then an unstable limit cycle appear at point HB_2_, at which time the waveform gradually evolves into state (III) and the waveform (IV) appears near LPC_1_.

**Table 1:** Model Paremeters

Symbol	Description	Value
*k*_0_	Feedback gain from magnetic flux	Scanned
*k*_1_	PY → PY coupling strength	1.8
*k*_2_	IN → PY coupling strength	1.5
*k*_3_	TC → PY coupling strength	1
*k*_4_	PY → IN coupling strength	4
*k*_5_	PY → TC coupling strength	3
*k*_6_	RE → TC coupling strength	0.6
*k*_7_	PY → RE coupling strength	3
*k*_8_	TC → RE coupling strength	10.5
*k*_9_	RE → RE coupling strength	0.2
*τ*_1_	PY timescale	26
*τ*_2_	IN timescale	32.5
*τ*_3_	TC timescale	2.6
*τ*_4_	RE timescale	2.6
*ε*_*py*_	Input PY	− 0.35
*ε*_*in*_	Input IN	− 3.4
*ε*_*tc*_	Input TC	− 2
*ε*_*re*_	Input RE	− 5
*ε*	Sigmoid steepness	2.5 × 10^5^
*α*	Linear intersection steepness	2.8
*β*	Linear intersection offset	0.5
*λ*_1_	Gain from PY	0.9
*λ*_2_	Self-inductance effect gain	0.5
*α*_1_	Constant parameter	0.4
*β*_1_	Constant parameter	0.02

## Discussion

Hundreds of millions of neurons are interconnected by synapses to form neural networks, and information is exchanged and propagated among neural networks through electrical signals [45–47]. Previous studies have proved that the complex electrophysiological state in the nervous system will inevitably produce electromagnetic fields, which will affect the electrical activity of neurons [48, 49]. However, it is not clear what kind of connection exists between electromagnetic induction and epilepsy. In this study, we found that electromagnetic induction affects the dynamic characteristics of epilepsy. In addition, our data show that the effect of the memristor is different for different pathways and models.

More and more evidence shows that network topology has a key influence on the collective behavior of neurons [12, 13, 50]. A previous study showed the importance of network topology for studying the spatiotemporal evolution of SWDs [12, 51]. In this study, we set up a single-compartment and coupled cortical thalamic model, and revealed by bifurcation analysis that the activation and attenuation of SWDs can be realized when the activation level of any neuron changes. In the coupled model, regulating *k*_*i*_ (*i* = 1, 2, 3, 5, 6, 7, 8, 9), the system appeared with more firing states such as fast spike wave oscillations, 2-SWDs, 3-SWDs, and Multiple spike wave discharges. These results are consistent with previous studies, indicating that the coupled model may trigger more bifurcation mechanisms and lead to the diversification of pathological states [28, 32].

As we all know, electromagnetic induction has a serious influence on collective behavior in neural networks [52, 53]. We found that the memristor showed a certain therapeutic effect on typical SWDs by adjusting *k*_*i*_ (*i* = 1, 2, 6, 9) in a single-compartment model. Specifically, electromagnetic induction makes the hopf point move, and the system transits to a larger monostable. Electromagnetic induction makes the stable point (HB) move, and the system transits to a wider monostable. Specifically, partial tonic discharge and rapid spike discharge of the system are reduced to normal discharge. Previous studies have reported similar findings, indicating that the presence of electromagnetic induction helps to inhibit the formation of absence seizures in the cortical thalamic system [54, 55]. Interestingly, in the pathway of cortical thalamus interaction (*k*_3_, *k*_5_, *k*_7_), the memristor fails. The pathological SWD oscillation state is aggravated due to more LPC bifurcations in the system to form a multistable region. In the coupled model, we also found that the existence of electromagnetic induction can also change the location and stability of Hopf bifurcation point for *k*_2_, *k*_7_ and *k*_8_. We focus on the inhibitory projection from RE-TC and RE–RE (*k*_6_, *k*_9_). Combined with the dynamic mechanism, 3,4-SWDs should be caused by torus bifurcation and multi-stable region of the system, while the slow wave oscillation with gradual distortion is mainly related to the position shift of Hopf bifurcation points and the inhibitory projection of RE–RE.

It should be noted that we limited the parameters and initial conditions of the memristor, and did not discuss the control strategy of the memristor, which is a limitation of our research. Secondly, the model we established is a simplified model in the macroscopic sense, and we should consider the basal ganglia and more nuclei in the future work.

## Conclusion

Based on our findings and previous studies, the theory that the coupled model can trigger more discharge states has been more effectively supported. Furthermore, previous studies have proved that memristor can be used as an additional strategy to treat epilepsy. However, in this study, we use bifurcation analysis to study the dynamic changes of multiple pathways in single-compartment and coupled models under electrical attraction. We observe that when the parameters of memristor are fixed, the choice of different brain regions may lead to the aggravation of the characteristics of absence seizures. Of course, this requires more clinical studies to test the theoretical results. We hope that our results can provide a testable hypothesis for the treatment of epilepsy patients in the future.

## Data Availability

All data generated or analysed during this study are available from the corresponding author upon reasonable request.

## Abbreviations

HB: Hopf bifurcations
LPC: Fold of cycle bifurcations
TR: Torus bifurcations
EEG: Electroencephalogram
SWDs: Spike and wave discharges
PY: Excitatory pyramidal neurons
IN: Inhibitory neurons
TC: Specific relay nucleus
RE: Thalamic reticular nucleus
